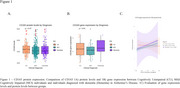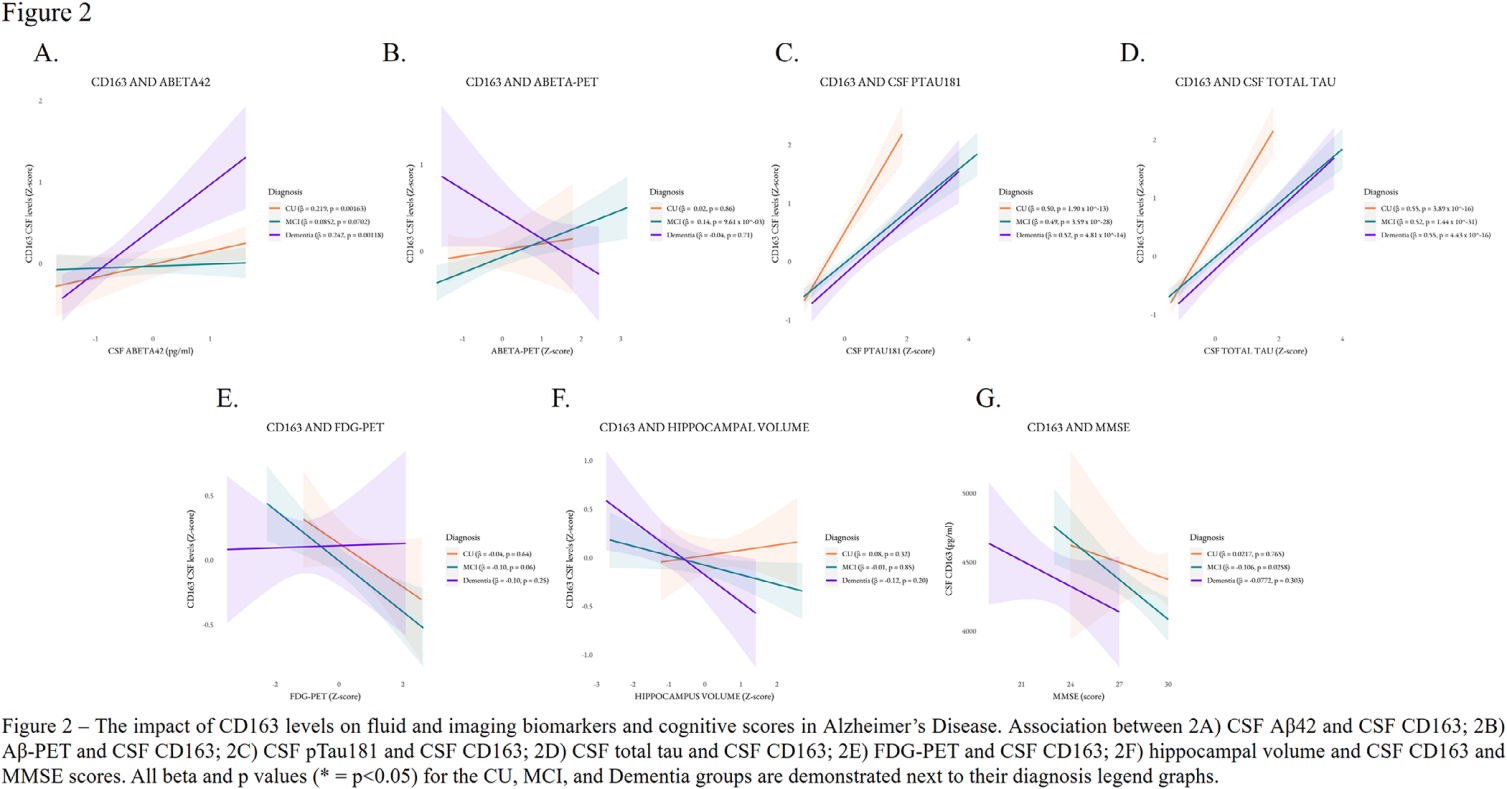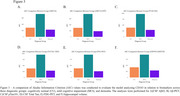# CD163 as a Predictive Prognostic Protein in Alzheimer's Disease

**DOI:** 10.1002/alz70855_106218

**Published:** 2025-12-24

**Authors:** Guilherme Schmitt Rieder, Gabriela Mantovani Baldasso, Christian Limberger, Marco Antônio De Bastiani, Diogo O. Souza, Débora Guerini de Souza, Eduardo R. Zimmer, João Batista Teixeira da Rocha

**Affiliations:** ^1^ Universidade Federal do Rio Grande do Sul, Porto Alegre, Rio Grande do Sul, Brazil; ^2^ Universidade Federal do Rio Grande do Sul, Porto Alegre, RS, Brazil; ^3^ Brain Institute of Rio Grande Do Sul, PUCRS, Porto Alegre, RS, Brazil; ^4^ McGill Centre for Studies in Aging, Montreal, QC, Canada

## Abstract

**Background:**

Neuroinflammation is a multifaceted response to brain injury, characterized by glial activation and the release of inflammatory mediators. Alzheimer's disease (AD) has a strong neuroinflammatory component in its pathophysiology. CD163 is a cellular marker of neuroinflammation, displaying increased expression in AD. CD163 encodes a transmembrane receptor primarily expressed in immunosuppressive cells, regulating inflammation and the immune response. However, its relationship with the core biomarkers in AD remains unclear. This study aims to evaluate the impact of the CD163 protein on astrogliosis, Aβ, and tau biomarkers in AD.

**Method:**

We included 170 cognitively unimpaired (CU), 411 mild cognitively impaired (MCI), and 138 individuals with dementia (Dementia) from ADNI with available fluid biomarkers, blood transcriptomics, and neuroimaging data at baseline. Generalized linear mixed models were performed in R to assess the association of CD163 protein levels with AD biomarkers, accounting for age and sex. Akaike Information Criterion (AIC) comparison was used to evaluate the model used to analyze CD163 as a function of biomarkers for each group.

**Result:**

No differences in CD163 protein levels and blood gene expression were observed between groups. In CU, total‐tau, Aβ_42,_ and pTau181 parameters were positively associated (β=0.55; 0.22 and 0.50) with CD163 CSF protein levels. In MCI, we found a positive association (β= 0.14; 0.49 and 0.52) between CD163 CSF levels with Aβ‐PET, pTau181, and total‐tau, as well as a negative association (β= ‐0.10.) with the MMSE score. In dementia, total‐tau, Aβ_42,_ and pTau181 parameters were positively associated (β= 0.55; 0.24 and 0.52) with CD163 CSF levels. No significant associations were found between CD163 CSF levels and hippocampal volume in the groups. The AIC values showed that individuals with dementia consistently present the lowest values, therefore, the correlations between CD163 and the biomarkers have less variability and greater precision for the dementia group.

**Conclusion:**

The biomarkers analyzed describe the dementia group more accurately; therefore, the positive correlation found between CD163 CSF levels and total‐tau, Aβ_42_, and pTau181 reinforces the role of CD163 as a potential indicator of advanced inflammatory and neurodegenerative processes. These results open new perspectives on the role of CD163 in AD.